# Supplementary material for: Linking high GC content to the repair of double strand breaks in prokaryotic genomes
Source: PLoS Genet. 2019 Nov 8;15(11):e1008493. doi: 10.1371/journal.pgen.1008493 (PMC6867656; doi:10.1371/journal.pgen.1008493)
Supplement: S1 Table — The formal model was GC = β0 + βKuKu + ∑i βitraiti + ϵ, where GC is genomic GC content and Ku is a binary variable representing the presence/absence of Ku. (PDF) [file pgen.1008493.s001.pdf]

S1 Table: Output of linear model relating GC content to environmental variables. The formal model was GC $= \beta_0 + \beta_{\text{Ku}}\text{Ku} + \sum_i \beta_i \text{trait}_i + \epsilon$, where GC is genomic GC content and Ku is a binary variable representing the presence/absence of Ku.

	β	P-value
(Intercept)	0.3267217383	2.44868893191596E-025
Ecosystem Category: Terrestrial	0.1101532163	3.38243170487937E-008
Ecosystem Type: Soil	0.0333399451	0.1077906646
Known Habitats: Soil	-0.0331866659	0.0551188689
Habitat: Terrestrial	0.1866541988	9.95695959696927E-025
Nitrogen Fixation	-0.0040711787	0.7635124534
Nitrogen production	-0.4157246148	3.66244214114883E-072
Facultative Anaerobe	0.0073411402	0.6375719405
Strict Aerobe	0.0859143088	7.46077194429656E-007
Strict Anaerobe	-0.0143781175	0.4307698204
Sporulation	-0.1033310344	3.56417677006438E-016
Hyperthermophilic	-0.0834952183	0.0006640906
Mesophilic	0.1342814922	5.81638646775348E-009
Thermophilic	0.2526565354	1.32824551249747E-020
Microaerophilic	-0.0631903302	3.51937747994195E-005
Psychrophilic	-0.2429898729	7.89192914664017E-037
Ku	0.0126640175	0.0042992051
