# Supplementary material for: Linking high GC content to the repair of double strand breaks in prokaryotic genomes
Source: PLoS Genet. 2019 Nov 8;15(11):e1008493. doi: 10.1371/journal.pgen.1008493 (PMC6867656; doi:10.1371/journal.pgen.1008493)
Supplement: S1 Fig — Note that some traits are highly correlated. (PDF) [file pgen.1008493.s002.pdf]

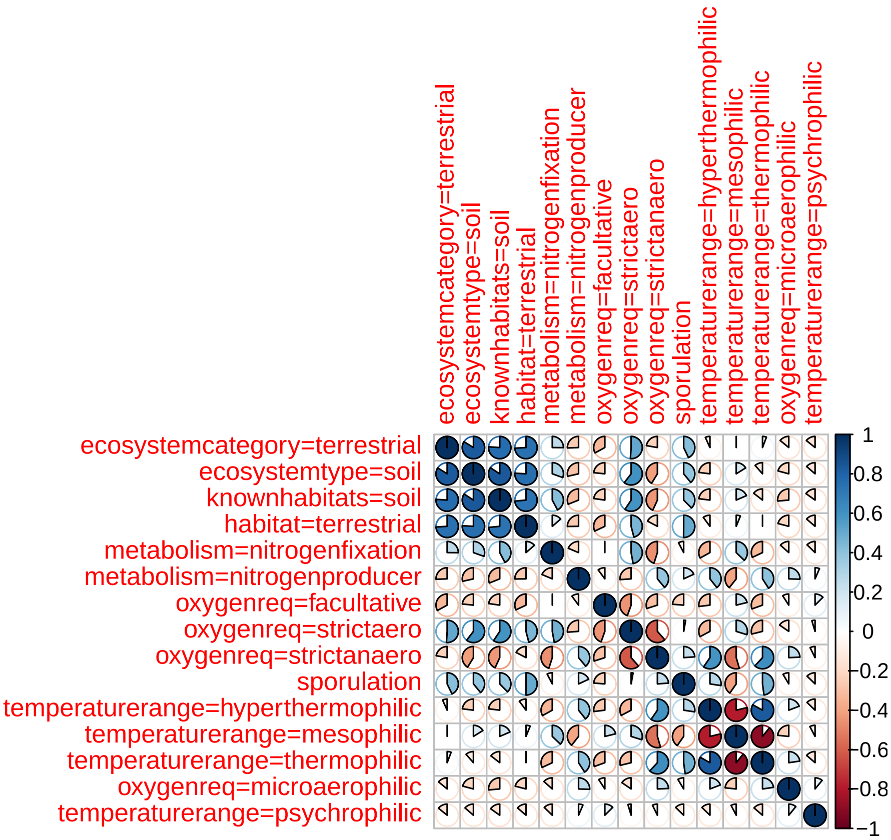

S1 Fig: The pairwise correlation between traits among species in the trait dataset. Note that some traits are highly correlated.
