# Supplementary material for: Linking high GC content to the repair of double strand breaks in prokaryotic genomes
Source: PLoS Genet. 2019 Nov 8;15(11):e1008493. doi: 10.1371/journal.pgen.1008493 (PMC6867656; doi:10.1371/journal.pgen.1008493)
Supplement: S2 Fig — Note that each point is an individual trait, as shown in Fig 1. The dashed diagonal line indicates the x = y line. For a direct analysis of the relationship between GC content and Ku incidence among organisms see Fig 2 and Table 1. (PDF) [file pgen.1008493.s003.pdf]

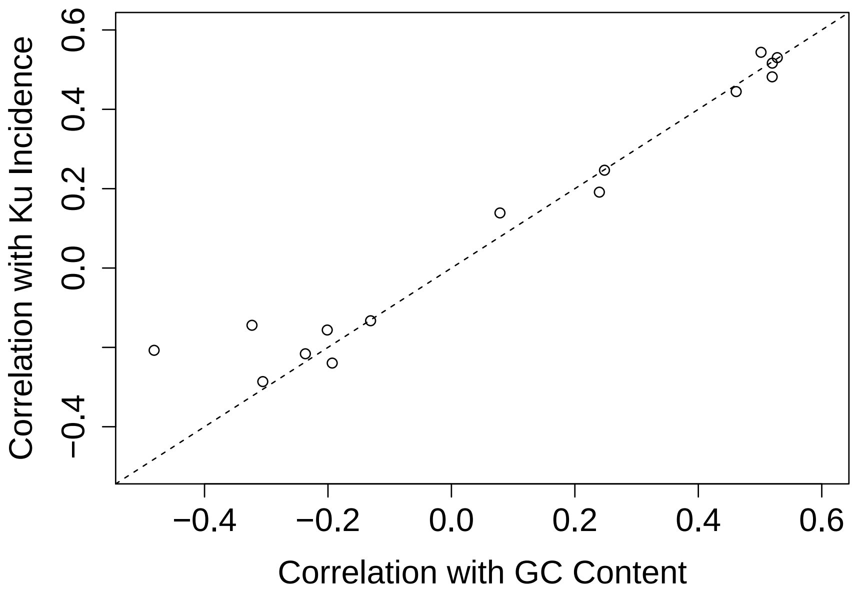

S2 Fig: The correlation of trait values for microbial species with their average genomic GC content is similar to the correlation of trait values with the presence/absence of Ku. Note that each point is an individual trait, as shown in Fig 1. The dashed diagonal line indicates the $x = y$ line. For a direct analysis of the relationship between GC content and Ku incidence among organisms see Fig 2 and Table 1.
