# Supplementary material for: Linking high GC content to the repair of double strand breaks in prokaryotic genomes
Source: PLoS Genet. 2019 Nov 8;15(11):e1008493. doi: 10.1371/journal.pgen.1008493 (PMC6867656; doi:10.1371/journal.pgen.1008493)
Supplement: S3 Fig — The effect of Ku is significant even taking phylogeny into account using an identical approach to overall genomic GC content (Table 1). (PDF) [file pgen.1008493.s004.pdf]

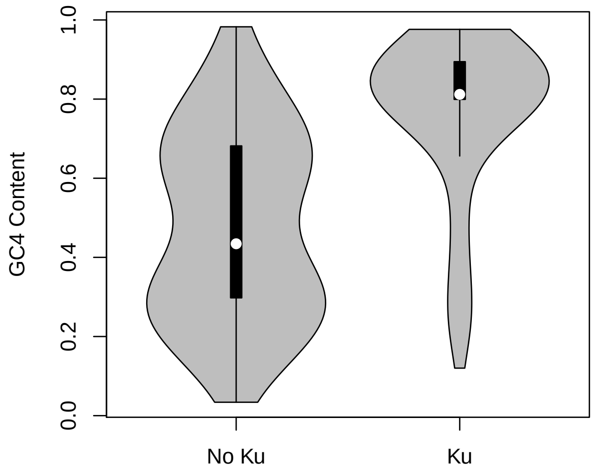

S3 Fig: GC content at fourfold degenerate sites follows a similar pattern to that of genomic GC content overall (Fig 2). The effect of Ku is significant even taking phylogeny into account using an identical approach to overall genomic GC content (Table 1).
