# Supplementary material for: Linking high GC content to the repair of double strand breaks in prokaryotic genomes
Source: PLoS Genet. 2019 Nov 8;15(11):e1008493. doi: 10.1371/journal.pgen.1008493 (PMC6867656; doi:10.1371/journal.pgen.1008493)
Supplement: S4 Fig — (a) Regression and contour lines were created using default ggplot settings (b) The positive GC versus Ku relationship hold across taxa, independently of any relationship with genome length. Regressions of GC versus log genome length for Ku and non-Ku genomes shown. (PDF) [file pgen.1008493.s005.pdf]

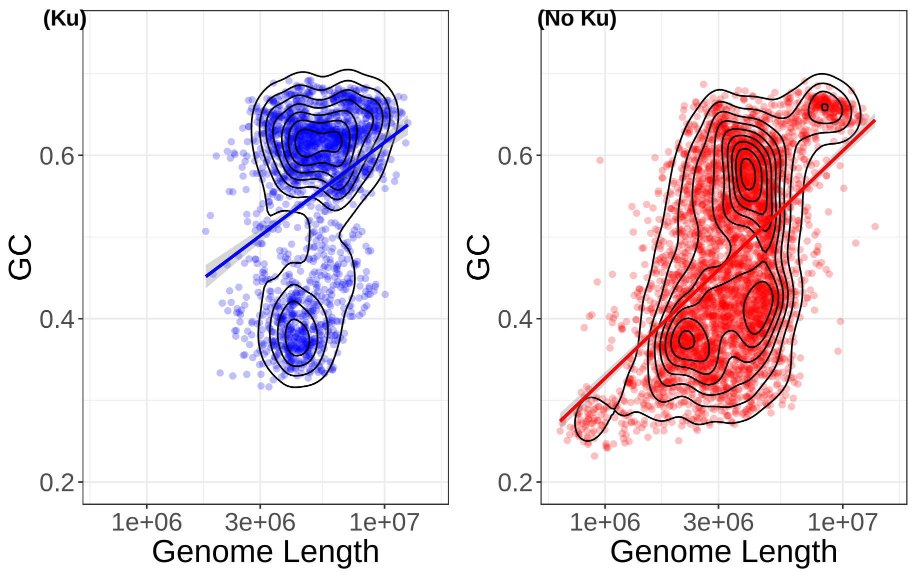

(a)

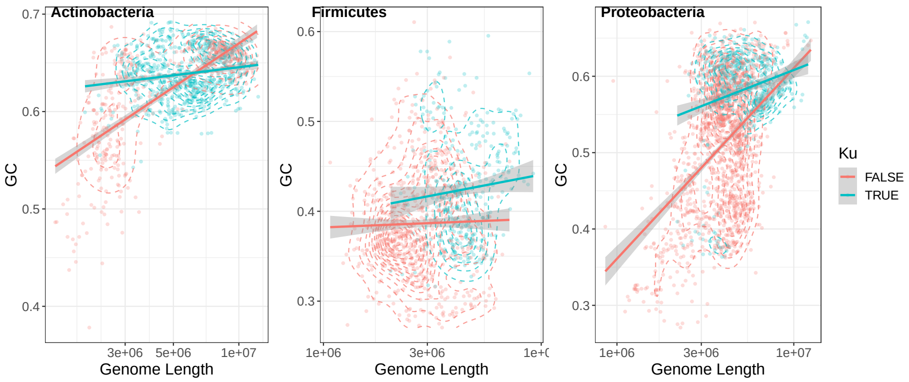

(b)

S4 Fig: While there is a positive GC content versus genome length trend, genomes with Ku have elevated GC independent of this relationship. (a) Regression and contour lines were created using default ggplot settings (b) The positive GC versus Ku relationship hold across taxa, independently of any relationship with genome length. Regressions of GC versus log genome length for Ku and non-Ku genomes shown.
