# Supplementary material for: Linking high GC content to the repair of double strand breaks in prokaryotic genomes
Source: PLoS Genet. 2019 Nov 8;15(11):e1008493. doi: 10.1371/journal.pgen.1008493 (PMC6867656; doi:10.1371/journal.pgen.1008493)
Supplement: S5 Fig — Organisms with the Ku protein did not differ significantly in their GC↔AT mutational biases from those without the Ku protein (t-test, p > 0.34). Estimates of mutational bias were obtained from Long et al. [6]. (PDF) [file pgen.1008493.s006.pdf]

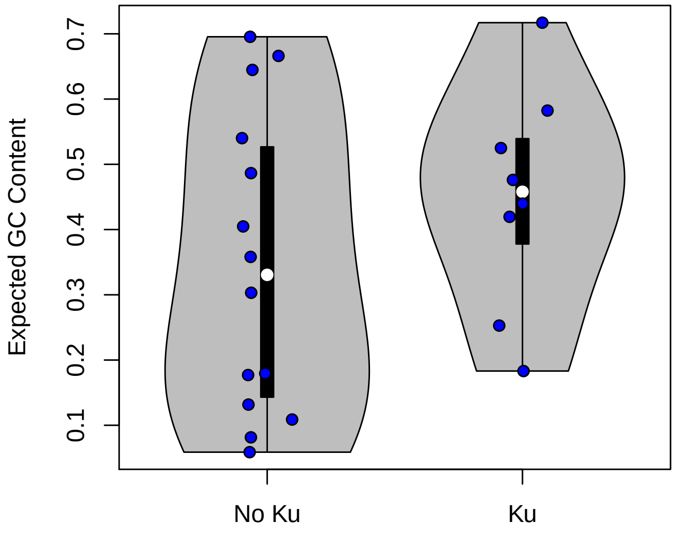

S5 Fig: Mutational bias does not appear to be associated with the NHEJ pathway. Organisms with the Ku protein did not differ significantly in their GC↔AT mutational biases from those without the Ku protein (t-test, $p > 0.34$). Estimates of mutational bias were obtained from Long et al. [1].
