# Supplementary material for: Linking high GC content to the repair of double strand breaks in prokaryotic genomes
Source: PLoS Genet. 2019 Nov 8;15(11):e1008493. doi: 10.1371/journal.pgen.1008493 (PMC6867656; doi:10.1371/journal.pgen.1008493)
Supplement: S6 Fig — (a,b) Genomes with Ku have, on average, even greater elevation of GC over expectation than genomes without Ku. Expected GC estimated from polymorphism data; in contrast to main text Fig 3, here we only use polymorphisms at fourfold degenerate sites. This signal is conservative due to observed polymorphisms experiencing some effects of BGC/selection (see Methods for discussion). (PDF) [file pgen.1008493.s007.pdf]

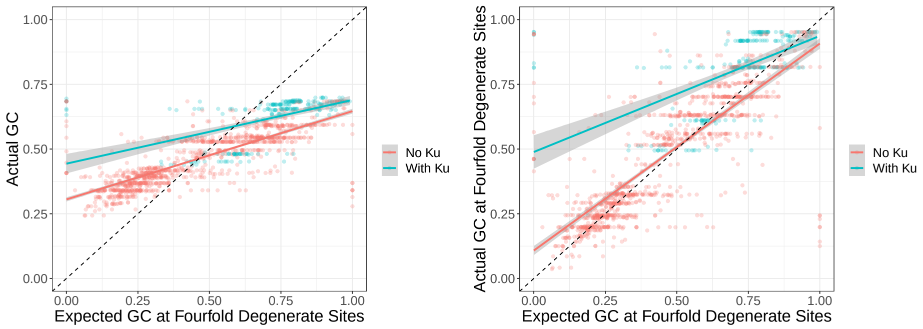

(a) (b)

S6 Fig: Genomes with Ku appear to fix GC alleles at a greater rate than expected (either due to BGC or selection). (a,b) Genomes with Ku have, on average, even greater elevation of GC over expectation than genomes without Ku. Expected GC estimated from polymorphism data; in contrast to main text Fig 3, here we only use polymorphisms at fourfold degenerate sites. This signal is conservative due to observed polymorphisms experiencing some effects of BGC/selection (see Methods for discussion).
