# Supplementary material for: Linking high GC content to the repair of double strand breaks in prokaryotic genomes
Source: PLoS Genet. 2019 Nov 8;15(11):e1008493. doi: 10.1371/journal.pgen.1008493 (PMC6867656; doi:10.1371/journal.pgen.1008493)
Supplement: S7 Fig — Genomes with Ku have, on average, even greater elevation of GC over expectation than genomes without Ku. This figure is identical to panels from Fig 3 and S6 Fig except that we draw loess smoothing lines using default ggplot settings instead of linear model fits. (PDF) [file pgen.1008493.s008.pdf]

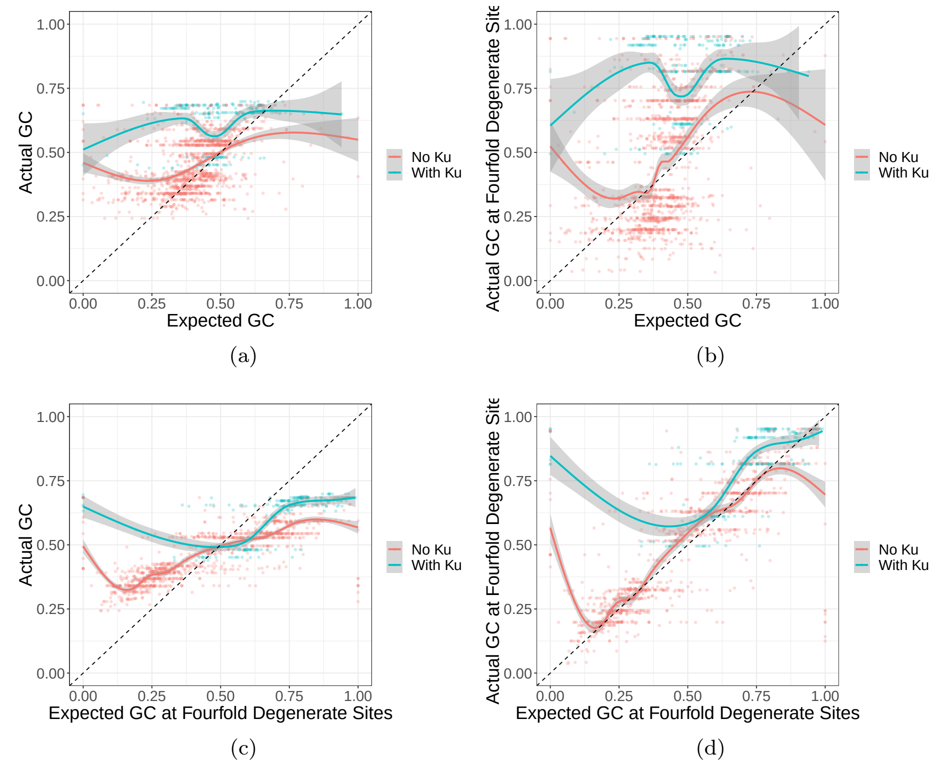

S7 Fig: Genomes with Ku appear to fix GC alleles at a greater rate than expected (either due to BGC to selection). Genomes with Ku have, on average, even greater elevation of GC over expectation than genomes without Ku. This figure is identical to panels from Fig 3 and S6 Fig except that we draw loess smoothing lines using default ggplot settings instead of linear model fits
