# Supplementary material for: Linking high GC content to the repair of double strand breaks in prokaryotic genomes
Source: PLoS Genet. 2019 Nov 8;15(11):e1008493. doi: 10.1371/journal.pgen.1008493 (PMC6867656; doi:10.1371/journal.pgen.1008493)
Supplement: S8 Fig — (a) Ku presence/absence plotted on the tips of the tree as in Fig 2 (blue with, red without Ku). (b) Ancestral state reconstruction of Ku (one rate class). Each internal node is represented by a pie chart describing the probability that that organism either had (black) or did not have (white) Ku. Notice that the root and most nodes near the root are likely to have had Ku. (PDF) [file pgen.1008493.s009.pdf]

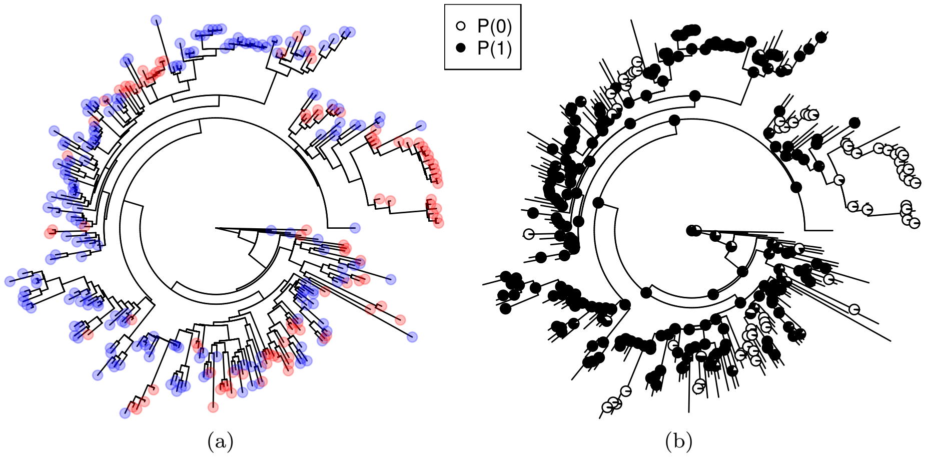

S8 Fig: Phylogeny of the *Baccilaceae* (subtree of the SILVA tree). (a) Ku presence/absence plotted on the tips of the tree as in Fig 2 (blue with, red without Ku). (b) Ancestral state reconstruction of Ku (one rate class). Each internal node is represented by a pie chart describing the probability that that organism either had (black) or did not have (white) Ku. Notice that the root and most nodes near the root are likely to have had Ku.
