# Supplementary material for: Linking high GC content to the repair of double strand breaks in prokaryotic genomes
Source: PLoS Genet. 2019 Nov 8;15(11):e1008493. doi: 10.1371/journal.pgen.1008493 (PMC6867656; doi:10.1371/journal.pgen.1008493)
Supplement: S9 Fig — (a) Estimated rate of recombination relative to mutation rate from Vos and Didelot [43]. (b,c) Estimated number of recombination events per gene family for species estimated with two methods by Rendueles et al. [44]. In general all of these methods give highly correlated results [44]. (PDF) [file pgen.1008493.s010.pdf]

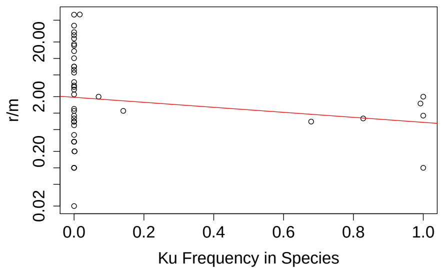

(a)

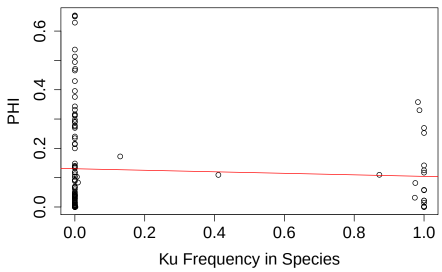

(b)

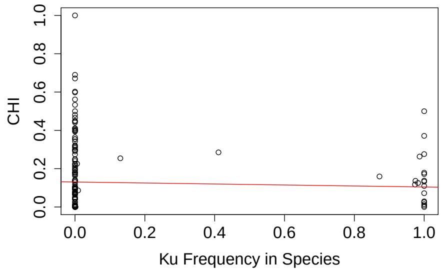

(c)

S9 Fig: Frequency of Ku presence does not appear to be positively associated with rates of homologous recombination for a species. (a) Estimated rate of recombination relative to mutation rate from Vos and Didelot [2]. (b,c) Estimated number of recombination events per gene family for species estimated with two methods by Rendueles et al. [3]. In general all of these methods give highly correlated results [3].
