# Supplementary material for: Linking high GC content to the repair of double strand breaks in prokaryotic genomes
Source: PLoS Genet. 2019 Nov 8;15(11):e1008493. doi: 10.1371/journal.pgen.1008493 (PMC6867656; doi:10.1371/journal.pgen.1008493)
Supplement: S10 Fig — We used the PHI statistic (see Methods) to determine if genes within each ATGC cluster of genomes had evidence for recombination. The percent of genes with evidence for recombination (out of all genes with sufficient data to test) showed no relationship to either Ku or GC content (averaged across genomes in a particular ATGC cluster). (PDF) [file pgen.1008493.s011.pdf]

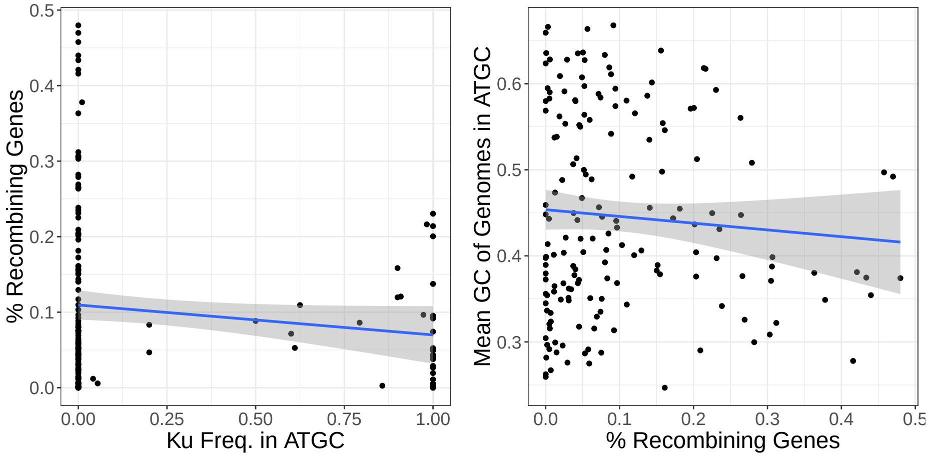

(a) (b)

S10 Fig: No relationship between genome-wide recombination frequency and (a) Ku incidence or (b) GC content in the ATGC database. We used the PHI statistic (see methods) to determine if genes within each ATGC cluster of genomes had evidence for recombination. The percent of genes with evidence for recombination (out of all genes with sufficient data to test) showed no relationship to either Ku or GC content (averaged across genomes in a particular ATGC cluster).
