# Supplementary material for: Linking high GC content to the repair of double strand breaks in prokaryotic genomes
Source: PLoS Genet. 2019 Nov 8;15(11):e1008493. doi: 10.1371/journal.pgen.1008493 (PMC6867656; doi:10.1371/journal.pgen.1008493)
Supplement: S11 Fig — Shown is the proportion of genomes within a species that have Ku (all RefSeq assemblies) plotted against the total number of assemblies in RefSeq for that species. (PDF) [file pgen.1008493.s012.pdf]

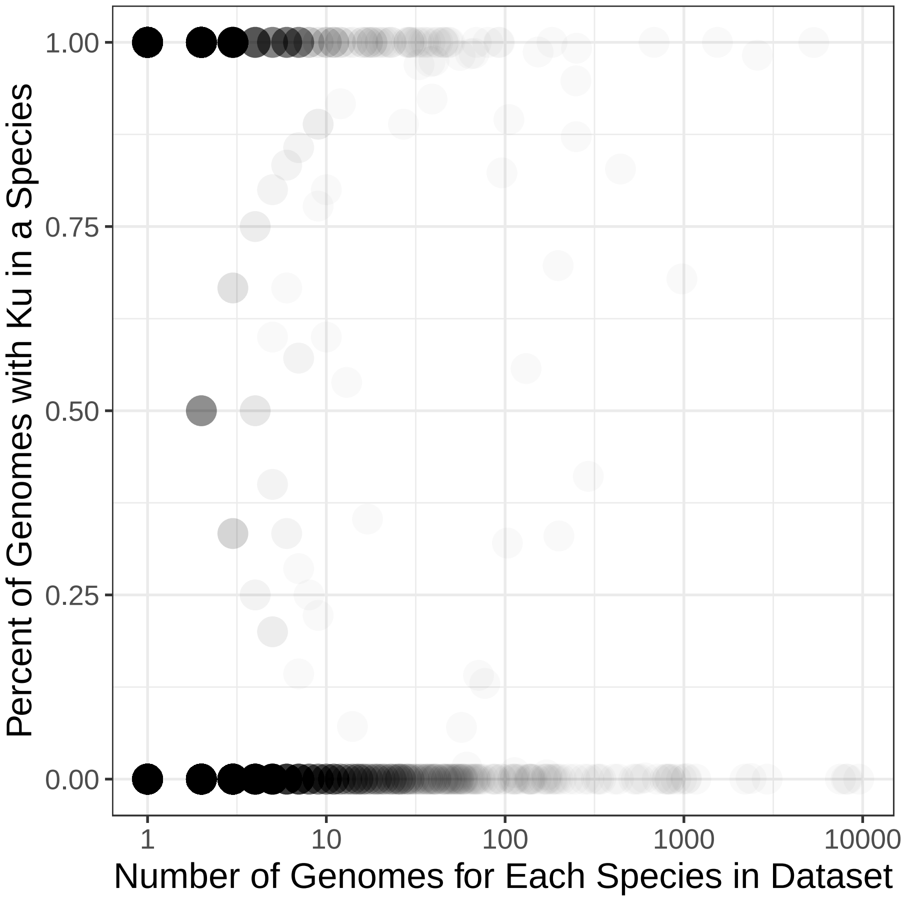

S11 Fig: Most species in RefSeq tend to always encode or always lack Ku on their genomes. Shown is the proportion of genomes within a species that have Ku (all RefSeq assemblies) plotted against the total number of assemblies in RefSeq for that species.
