# Supplementary material for: Linking high GC content to the repair of double strand breaks in prokaryotic genomes
Source: PLoS Genet. 2019 Nov 8;15(11):e1008493. doi: 10.1371/journal.pgen.1008493 (PMC6867656; doi:10.1371/journal.pgen.1008493)
Supplement: S12 Fig — (a) Estimates based on all polymorphisms. (b) Estimates based on polymorphisms at fourfold degenerate sites. Here we see selection/BGC appears to bias the polymorphism estimates when mutation is extremely biased towards AT. (PDF) [file pgen.1008493.s013.pdf]

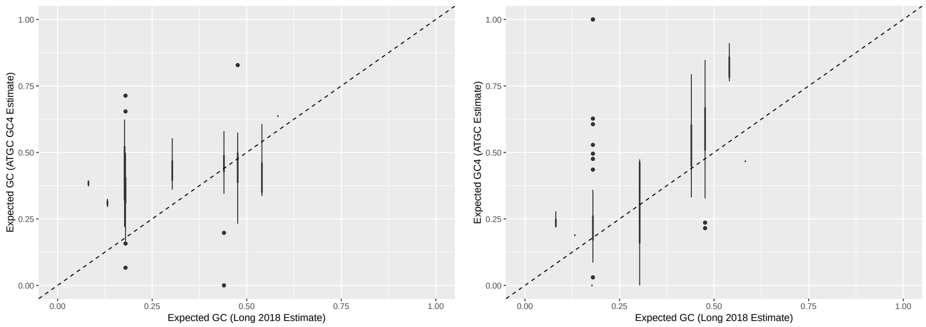

(a) (b)

S12 Fig: We evaluate the use of polymorphisms as a proxy for mutation by comparing estimates for the few species present in both the polymorphism and mutation accumulation data. (a) Estimates based on all polymorphisms. (b) Estimates based on polymorphisms at fourfold degenerate sites. Here we see selection/BGC appears to bias the polymorphism estimates when mutation is extremely biased towards AT.
