# Supplementary material for: Linking high GC content to the repair of double strand breaks in prokaryotic genomes
Source: PLoS Genet. 2019 Nov 8;15(11):e1008493. doi: 10.1371/journal.pgen.1008493 (PMC6867656; doi:10.1371/journal.pgen.1008493)
Supplement: Attachment — Submitted filename: ResponseToReviewers_PlosGen.pdf [file pgen.1008493.s014.pdf]

Editor:
Thank you very much for submitting your Research Article entitled 'Linking high GC content to the repair of double strand breaks in prokaryotic genomes' to PLOS Genetics. Your manuscript was fully evaluated at the editorial level and by independent peer reviewers. The reviewers appreciated the attention to an important topic but identified some aspects of the manuscript that should be improved. In particular, reviewer 3 makes suggestions for inclusion of additional data on homologous recombination, and use of the PHI test as in previous comparable studies, and we would like to encourage the authors to follow these suggestions.

We thank the editor for their consideration of our manuscript. See below for specific responses to reviewer comments as well as detailed changes to the manuscript. Specifically we would like to call the editor's attention to Figure 4 and S10 Fig which address Reviewer #3's suggestion that we apply the PHI test to our dataset. Also see additional analyses in S1, S8, and S11 Figures and described in response to specific reviewer requests below.

We have also made our intermediate datasets and code available: https://github.com/jlw-ecoevo/gcku

Reviewer #1: In this study, Weissman and colleagues explore a new mechanism potentially explaining the evolution of GC-content in bacteria. Many works have been published on this question, but yet, no single explanation has imposed itself. The authors hypothesize that DNA breaks and repair could be the underlying cause of GC-content evolution across bacteria. Although they cannot provide direct evidence supporting their hypothesis, the results show, at the very least, a clear link between NHEJ and GC-content. The methods used by the author are generally sound and only have a few criticisms. The manuscript is well written and very pleasant to read. I think this is an interesting hypothesis and a good study. As mentioned by the authors, future experimental works could potentially test this hypothesis.

The methods appear adequate as far as I can judge, and I don't have any major concerns. However, I am not sure to understand how the authors calculated the incidence of Ku across the data set. For what I understood, it represents the probability that Ku is really present in a genome. Figure S1 is particularly intriguing, but that I am not sure what to get from it. It seems to represent a correlation of correlation coefficients, which might be a very indirect way to show a correlation. It would be more straightforward to represent the correlation between Ku incidence and GC-content directly.

We thank the reviewer for their comments and apologize for any confusion here. We searched each genome against a hidden Markov model provided by the Pfam database for the Ku protein, using the program hmmer. This is a standard method to search for protein homologs in a set of sequences, and is widely used in the literature. In practice, this is similar to running a BLAST search, though is far more sensitive in terms of detecting distance protein homology. Like BLAST, hmmer returns an evalue for each search, essentially the probability that a given sequence is a homolog of the protein family in question (corrected for the size of the database being searched). It is standard to apply a cutoff evalue (similar to a threshold for significance) to classify genomes as having or lacking a given gene (with some inevitable rate of false positives).

In the case of Fig 1, where we associate traits with Ku and GC, we have trait values at the species level. Therefore, we sampled a single genome from RefSeq for each species. As the reviewer notes, this is essentially a point estimate of the probability that a member of a given species has Ku. In

general, our sampling should not be an issue since Ku tends to be uniformly present or absent within a species. We have clarified this point in the methods (lines 385-390) and added S11 Fig:

"To assess trait vs. Ku relationships we sampled a single genome per species from our RefSeq dataset to determine Ku presence/absence in species with trait data available (617, 2062 without). Most species either always have or always lack Ku (S11 Fig), meaning that sampling should give a reliable estimate of whether we can expect a species to typically have Ku."

With respect to S1 Fig (now S2 Fig), this was meant simply to quantify the agreement apparent in Fig 1 with respect to trait values. The relationship between Ku and GC content is, in fact, shown in Fig 2a (using all genomes in RefSeq rather than only the subset for which we had trait data). We have modified the legend of Fig S2 to clarify:

"The correlation of trait values for microbial species with their average genomic GC content is similar to the correlation of trait values with the presence/absence of Ku. Note that each point is an individual trait, as shown in Fig 1. The dashed diagonal line indicates the x = y line. For a direct analysis of the relationship between GC content and Ku incidence among organisms see Fig 2 and Table 1."

and we have also noted the correlation between Ku and GC across all genomes in the main text (lines 104-107):

"Using a large set of genomes from RefSeq we found that genomes with Ku have a dramatically shifted GC content relative to genomes without Ku (Fig 2A, S3 Fig; Pearson correlation between GC content and Ku across genomes, $r = 0.54$, $p < 2.2\times10^{-16}$)"

The main issue with the results of the manuscript is that the authors are using the presence of Ku as evidence for more frequent DSBs. As stated by the authors, the NHEJ pathway is either present or absent but DSBs can occur at different rates. Figure 2A is rather convincing but it would be interesting to indicate the size of each sample. Also, it is not clear to me whether the data were computed on the entire dataset of genomes or if only one genome were selected for each species.

We agree with the reviewer (and discuss in the main text, as the reviewer notes) that Ku presence/absence is an imperfect measure of the rate of DSB formation an organism experiences. That said, we do expect that NHEJ will be favored in specific environments (e.g., Fig 1), and it's widespread but sparse distribution makes it a promising indicator of frequent damage. We hope to motivate future work that surveys the rate of DSB formation directly in the environment, though as we note in our conclusions this is a non-trivial task.

We also thank the reviewer for pointing out a part of the analysis that was inadequately described. While the sample size was indicated in the methods (21389 out of 104297 genomes with Ku), it was not specified on the actual figure. We have modified the legend of Fig 2 to reflect this:

"The relationship between genomic GC content and the NHEJ pathway in prokaryotes. (a) Microbes that code for the Ku protein tend to have much

higher genomic GC content than those that do not (all RefSeq assemblies shown, 21389 out of 104297 genomes encode Ku).”

Species that don’t encode Ku appear to present a wider range of GC-content while species that encode Ku appear much more biased toward high GC-content. It would be informative to explore and discuss the rare species that encode Ku but present a relatively low GC-content. These cases might be insightful, but maybe the authors did not find anything worth reporting in the manuscript.

It is true that Ku-encoding organisms have a much narrower range of GC content than Ku-lacking organisms. This is partially attributable to the fact that there are many more Ku-lacking organisms in the dataset. At the same time, we might expect this pattern for other reasons. Specifically, while the presence of Ku might indicate environmental conditions with high damage and low growth, the lack of Ku does not necessarily indicate low rates of damage. Organisms without Ku but still experiencing high rates of DSB formation would still be expected to have high GC content under our hypothesis. We make this point briefly in our conclusions (lines 352-357):

“While the presence of NHEJ cannot single-handedly explain high GC content in all organisms (there are many organisms incapable of NHEJ that still have high GC content, Fig 2), it is possible that DSB formation can (or at least come close). For example, Deinococcus radiodurans is resilient to extremely high rates of DSB formation [61] and has high genomic GC content, but lacks Ku.”

The reviewer makes an interesting point about the organisms encoding Ku but with low GC content. In fact, 80% of these genomes come from the *Baccilaceae*. This family typically has low GC content (>99% of genomes in RefSeq have <50% GC, and 76% have <40% GC), and an ancestral state reconstruction suggests that the MRCA of the group had Ku (which has been lost multiple times). We have added these analyses to the manuscript (lines 170-178) along with S8 Fig:

“Finally, we note that there is a small subset of genomes in Fig 2 that both encode Ku and have a low GC content (< 40%). Of these, 80% belong to the family Baccilaceae. This family has uniformly low GC content (> 99% of genomes have GC content < 50%, and 76% have GC content < 40%), and an ancestral state reconstruction suggests that its most recent common ancestor encoded Ku (S8 Fig and see methods), though Ku has been lost multiple times across the group. We do not know why the Baccilaceae violate the pattern seen across the rest of the dataset; it may be an accident of evolutionary history or some particular aspect of this group's ecology and/or physiology.”

and (lines 422-429):

“We performed an ancestral state reconstruction of the presence/absence of Ku in the Baccilaceae (S8 Fig). We used the R package corHMM to reconstruct the evolutionary history of this trait on the subtree of the SILVA phylogeny describing the Baccilaceae [67]. We allowed for up to two rate classes (for trait evolution) across the tree when building our evolutionary model (rate.cat parameter in function corHMM, otherwise default parameters), but found that

a model with a single rate class had a lower AICc (257.3119 vs. 263.8347). Thus we only retained a model using a single rate class across the tree.”

The authors argue the restriction systems might elevate the frequency of DSBs and use the presence of RM systems as an indirect indicator for elevated DSBs. If we follow their logic, we might expect species encoding larger numbers of RM systems to present higher GC-content. I find this argument not very convincing considering that Helicobacter pylori encodes an exceptionally high number of RM systems (Oliveira, Touchon and Rocha, NAR 2014) but present a relatively low GC-content (~39%).

This doesn’t necessarily follow, since even genomes with an exception number of RM systems are likely to potentially target only a small proportion of the genome. It would be possible to see a local effect of RM systems, which have very specific target sites, without seeing much overall signal for high GC across the genome. The very specificity of these systems constrains the magnitude of these effects (Especially since we see that often target sites themselves are selected against). We now note this in the main text (Lines 323-329):

“Finally, we caution that one is unlikely to see genome-wide differences in GC content when comparing across organisms with different numbers of restriction enzymes, since restriction sites comprise a very limited subset of loci along the genome (and self targeting should be somewhat restrained via methylation of the host chromosome). Presumably if self targeting was frequent enough to select for elevated GC content at a genome-wide scale, the corresponding cost of encoding these enzymes would be prohibitively high.”

This is similar to the logic described in Lassalle et al. [7] that you might see correlations between recombination rate and GC content along a genome, but not within genomes.

Finally, I think it is interesting that GC-content and genome length correlate. This observation is not new, but it supports the hypothesis of the authors. I believe it can be safely assumed that, overall, bacteria with larger genomes endure more frequent DSBs. Under the authors’ assumption, the higher GC-content of larger genomes could be explained by the need to repair more frequent DNA breaks. I think this was not explicitly formulated in the manuscript and could be emphasized.

We generally agree with the reviewer and, in fact, have made this point in a previous version of this manuscript (submitted to another journal). Unfortunately a different reviewer took issue with this point previously, and since we deemed it non-essential to our overall message we opted to remove it. Specifically, they questioned the assumption that larger genomes are more prone to breaks, and suggested that the rate of DSB formation would have to scale super-linearly with genome size to see this type of effect (though we suspect that this intuition may be incorrect, since all breaks must be repaired before replication can proceed and thus are likely felt on a per-genome rather than a per-base scale). Nevertheless, since we do not know of any datasets describing how the rate of DSB formation scales with genome size, we are wary of making this point in the paper. It occurs to us that we already speculate about mechanisms to a degree some readers may find excessive, and we think it may be wise to restrain ourselves here (since this is a minor, though interesting, point).

Reviewer #2: I have reviewed this article before for another journal. In this new version, most of my initial critics have been addressed and I find the article quite good. The relationships between genomic GC-content and the presence of the NHEJ pathway is an interesting point to bring to the debate on the

evolution of GC-content in genomes. However, I still have difficulties with the hypothesis of the authors that there is selection for high GC-content to favor double strand breaks repair. It is not clear to me why the hypothesis that NHEJ is itself a repair mechanism that is biased toward GC (just like BGC in HR) is not considered and discussed. The argument that GC-rich regions are better repaired seems relatively weak to me.

We thank the reviewer for spending multiple rounds of review on our manuscript. We do feel that the work has substantially benefited from previous review.

There is an important distinction here between the mutation-generation process and the fixation process. The data (fig 3) shows that GC alleles are fixed at a higher rate than AT alleles (not simply produced at a higher rate). If Ku repair favored the creation of GC alleles, we would see a mutational rather than a fixation bias among Ku-favoring organisms. BGC is slightly different since it is not producing novel GC alleles, but rather is increasing their rate of spread across a population. We have clarified this in the main text (lines 162-169):

"Thus, the association between Ku and genomic GC content is not due to differences in mutational bias. This implies that DSBs are either leading to selection for high GC content or influencing the rate and/or biases of homologous recombination to increase the overall action of BGC. We emphasize that this e#ectively rules out the possibility that biases during NHEJ repair are causing the observed patterns. NHEJ repair may be error-prone, but if those errors (i.e., mutations) were driving genome-wide GC-bias it would affect the GC-bias of polymorphisms as well as fixed alleles in the test described above."

We readily admit that we do not have direct evidence for our repair hypothesis. Nevertheless, we think this hypothesis is particularly useful as it proposes a specific mechanistic basis for selection on GC content across the entire genome that can be directly probed with experimental approaches.

Reviewer #3: This manuscript presents new observations and a new hypothesis to explain the long-time puzzle of prokaryotic GC content heterogeneity, and the discrepancy between observed GC contents and their – almost universally lower – expected value based on mutational patterns. They report that the non-homologous end-joining (NHEJ) protein Ku is strikingly associated with high genome GC content, and also with the departures from mutational equilibrium, in a stronger way than any previously considered trait (notably those associated to lifestyle). The authors interpret this Ku-GC association as a signature of GC elevation being a response to frequent exposure to double-stranded DNA (dsDNA) break (DSBs). This is considered under several hypotheses, including that GC elevation and Ku occurrence may both be correlated responses the high incidence of DSBs, via separate mechanisms. Alternatively, they investigate a hypothesis where Ku is causally linked to GC elevation, via selective process promoting the elevation of GC content in the genome and in particular in regions susceptible to regular DSBs such as self-target sites for restriction enzymes to improve the efficiency of Ku repair function. They conclude that Ku (or the NHEJ pathway) is unlikely to account on its own for the whole higher-than-expected-GC phenomenon, but may at least be the functional mechanism of a selective process that accounts for part of this phenomenon.

The manuscript is very well written and documented, and presents relevant analyses to test the new hypothesis. The authors also attempt to link these new results to observations made previously regarding other hypotheses of mechanism for above-mutational-equilibrium genome GC contents,

namely selection for higher %GC per se, and biased gene conversion (BGC).

The evidence presented in support of the Ku-GC association is sufficient and convincing, and its interpretation is cautiously discussed to consider known evidence, and to take into account potential interactions or confounding signatures with other mechanisms.

However, it would be desirable that the authors bring their study a step further, and bring a bit more material to help the reader (and future investigations) to resolve this puzzle. Namely, in order to test the relevance of the BGC hypothesis in the light of the facts presented in this study, they confront Ku occurrence and GC content data are to homologous recombination (HR) data, which are only recovered from other studies. This brings the concern that these data are not properly matched with the sty's own datasets: summary statistic from studies using different genome sets (and potentially different set of sequences within genomes) on the basis of the sole species name is unlikely to reflect the exact properties of the genome datasets investigated here. Considering the scale of the present investigation (the whole prokaryotic tree of life), it is crucial that each data point be accurately representing the properties of the considered organism, and hence that all measurements be made on the same dataset. As explained below, applying the HR test/quantification procedures described in the cited literature to this dataset would be a feasible undertaking, and would add much value to the paper.

Finally, I notice that the intermediary data is not made available. This includes tables describing the sets of genomes used, the occurrence of Ku in these genomes, the list of restriction enzyme found in them and their corresponding target sequences, the genome tree presented Fig 2 in machine-readable format, the estimates of GC at the mutational equilibrium, etc. the scripts used to generate such data, as well as those used to test their association, should be provided as well. I think that publication in scientific journal, and especially in the open-access pioneer PLoS journals, should always be backed by full access to data and proceedings of the analyses so they can be replicated. Please attach them as a supplement, or provide a link to an external data/code repository (my recommendation).

I let the editor appreciate the relevance of the request for additional data on HR. Provided that the few minor comments below are addressed and that intermediary data are provided, I think the manuscript would be otherwise generally fit for publication in PLoS Genetics. I thus recommend the paper for minor revision.

We thank the reviewer for their in-depth comments. As detailed below, we have incorporated an analysis of recombination using the PHI statistic as requested (which nicely complemented our other analyses and resulted in the addition of a figure to the main text – Fig 4). We have also made our code and intermediate datasets available on github: https://github.com/jlw-ecoevo/gcku

Detailed comments

L70-82: this paragraph belongs to the introduction, with which it is slightly redundant.

While we appreciate that there is some redundancy here, we think having a bit of motivation at the outset of our Results and Discussion section helps adequately frame the section and guide the reader. Additionally, we feel this context is important for readers who tend to read papers out of order. Since this is a combined Results/Discussion section we do feel it is appropriate to have some discussion of previous work here. As this is a matter of style rather than substance, we are opting to go with our gut in this specific instance and keep the paragraph as is.

L87-89: this correlation of the Ku and GC, as revealed by correlation of each with 'third-party' trait, is striking. However, it would be nice to have a more straightforward estimate and visualisation of their association. Could the authors provide a correlation r^2 and p-value for GC ~ Ku occurrence? In complement of the PCA in fig. 1, could they also plot the result of a linear discriminant analysis (LDA) maximizing the separation of the samples based on their Ku +/- state, and plotting the %GC over it (as well as showing the explained variance of such a projection)?
Actually, something like a heatmap of a correlation matrix of all these traits would be helpful (in supplement) for the reader to see how the traits are associated with each other.

We agree that such information will be useful to our readers. We now note the correlation between Ku and GC across all genomes in the main text (lines 104-107):

"Using a large set of genomes from RefSeq we found that genomes with Ku have a dramatically shifted GC content relative to genomes without Ku (Fig 2A, S3 Fig; Pearson correlation between GC content and Ku across genomes, $r = 0.54$, $p < 2.2 \times 10^{-16}$)"

We also have added S1 Fig that describes the pairwise correlation between traits as requested.

With respect to the recommended LDA analysis, we do not understand quite what the reviewer is asking for. In addition, we are worried that many redundant analyses of the trait data may confuse a reader and dilute the overall message (especially since our more central and robust analysis of the GC-Ku relationship comes in the next section – the trait analysis was meant mostly to motivate downstream analyses).

L90-92 / S1 Table: I think that the table legend should spell out how the model was formulated (like give the R code or a more formal string like 'y ~ trait1 + trait2'). Once that is clarified, it would be interesting to present results of a general linear model where the prioritization of would have been different: with Ku as first explanatory variable, would the other traits have any variance left to explain?

We have modified the S1 Table legend to include the model formula.

With respect to the reviewers other request, we are not entirely sure what is meant. When including all variables in the model both Ku and many traits have significant p-values, meaning that all of these variables explain some of the variance even in the presence of the others (so in response to the above question: yes). This is also stated in the main text (lines 93-98):

"Nevertheless, the inclusion of Ku along with ecological traits in a linear model to explain genomic GC content resulted in most other environmental traits still being statistically significant (S1 Table), indicating that either there is some aspect of the environment affecting GC content that is not attributable to DSBs or that NHEJ is an imperfect indicator of the rate of DSB formation (or both)."

L95-96: "In fact this is trivially true, as Ku presence is a discrete, binary variable whereas the rate of DSB formation is continuous."
This is a relevant point, and should be considered further. In fact, the presence/absence of the Ku protein (used as a proxy of a functional NHEJ pathway) is a trait that can vary among strains of a clade or species, as stated by the authors L176-178. Transitions between the Ku +/- states might have

happened recently in certain strain lineages, and at potentially high frequency over time. On the contrary, %GC increase is expected to be a long process, given that the effect size of either selection for higher %GC or BGC phenomena are likely small, that they act against the mutational bias, and that selection for other traits may interfere with this background amelioration process. This is to be opposed to phenotypic traits (usually considered for correlation under BM or OU models) that result from the expression of the genotype of an individual organism, i.e. in sync with its current genotype.
It follows that the association between a potentially recently acquired trait (Ku presence) and the result of a long-standing process (%GC increase away from the mutational equilibrium) could possibly be coincidental. The authors should try and repeat their analyses by restricting them to genomes in clades where the Ku +/- state is conserved, and where we can expect that it has been present/absent for long enough so that the base substitution process is in its steady state. The situation that "Ku presence/absence is sprinkled throughout the prokaryotic phylogeny", and described in Figure 2B, where it seems that many clades have a homogeneous pattern of Ku occurrence, should allow them to run such restricted analyses with enough statistical power (while still using the phylogenetically-aware regression models to avoid over-counting the replicated data points within such homogeneous clades). This is an important point, as most studies trying to confirm/invalidate the hypothesis of BGC have tried to correlate the %GC with the recombination rate inferred from recent polymorphism data, which again reflect a recent property of the population, but might not reflect the long-term average recombination rate that the lineage has experienced – a major issue that prevented most past analyses to settle the debate on the existence or not of BGC in Prokaryotes. Ku occurrence is a simple binary trait and its past distribution is more easily estimated than the past recombination rate, which estimation from polymorphism data is inherently biased towards recent times due to saturation of homoplasy signals; by studying this simpler trait, the authors here have an opportunity to bring stronger evidence on that subject than any other previous study.

We thank the reviewer as we had not considered this type of analysis previously. We performed the requested analysis (lines 416-420):

"Finally, for our "Uniform Ku" models we excluded all genera from our dataset that had fewer than two genomes with which to assess Ku incidence, and then excluded any genera for which Ku incidence was not uniform (all genomes had Ku or all genomes lacked Ku). We then repeated our above analysis (779 taxa with Ku, 2365 without)."

and found that our results were qualitatively unchanged (lines 117-121):

"Finally, to control for the possibility that Ku gain/loss via horizontal transfer is frequent and potentially confounding, we also restricted our analysis to a subset of the data where Ku presence/absence did not vary within each genera (discarding variable genera) and found qualitatively the same result (Table 1)."

Section "No Apparent Relationship Between Rate of Homologous Recombination and NHEJ":
I agree with the general conclusions of the authors for this section, that is the impossibility to conclude given the data, but I think they could try and provide further evidence to fuel the debate. In particular, they only rely on data from previous study to quantify the effect of homologous recombination (HR) on species they investigated in their own dataset. The third-party data they report is likely to be inadequate to answer the question asked, for several reasons.
The quantification of HR rates (r/m) by Vos and Didelot (ref [44]) is made using ClonalFrame, a

method that is able to grasp the long-term average HR rate (see comment above), which is a good thing, but was based on multi-locus data and on quite a variable set of strains depending on the species, thus unlikely to reflect findings from sets of whole-genomes of calibrated diversity (from the ATGC database) used in the present study.
The data from Ruendules et al. [45] are also unlikely to have used the same set of genomes, and use simple linkage disequilibrium-based metrics which have been designed to perform test of occurrence of HR, not to quantify it, and which application at the whole-genome scale is unlikely to grasp any nuance in such signal.
The fairer comparison is with the data from Lassalle et al. (ref [7]), but again the genome datasets are unlikely to be matched. Published genome data expand rapidly and, as a consequence, prokaryotic species definitions are being regularly revised; the genomes available for what was considered to be B. anthracis by Lassalle et al. in 2015 is thus unlikely what is available today in ATGC database under this same name. I believe this drastically limits the scope of what the authors are able to say about HR in the framework of this study.
I would suggest that the authors replicate the procedure used by Lassalle et al., that is running the PHI test on the core gene alignments of their species datasets (or at least a representative subset), as provided by the ATGC database. The PHI test is very fast and can easily be ran in parallel on a large collection of gene alignments. This is not essential to the core argument of the paper, but would help going further on the matter.

We thank the reviewer for pointing out a potential issue with our analysis. Nevertheless, since Ku incidence tends to be relatively uniform within a species (S11 Fig) this is something of a moot point (this can also be seen in S9 Fig and S10 Fig as most of the points lie at the extremes of the x-axis). Nevertheless, we agree it is important to be sure that our data is consistent. As requested we ran the PHI test (S10 Fig) and found nearly identical results to those using separate datasets (S9 Fig), (lines 187-192):

***"We saw no positive as-
sociation between Ku incidence and inferred rates of homologous recombination looking between genomes, as would be predicted by this hypothesis (S9 Fig with data from [44, 45], and S10 Fig with data from the ATGC database [43]). In fact the relationship appeared to be negative regardless of method to measure recombination rate (though not significant)."***

The idea to replicate the within-genome analysis of Lassalle et al. using the ATGC data is an interesting one, and led to some results that we think nicely complement our other analyses (so much so that we have added a figure to the main text – Fig 4). We found some evidence that could by taken as supporting BGC in general (though see Bobay and Ochman [10] for why this type of evidence is flawed), but that rejects any link between BGC and the Ku-GC pattern we observe (lines 215-228):

"Given the small number of organisms in Lassalle et al.'s dataset that had Ku, we endeavoured to repeat this analysis using a larger set of organisms. Using the ATGC database (as we did with our analysis of polymorphism earlier), we obtained multiple alignments of all orthologous genes for each cluster of organ-isms [43]. We then classiffed genes as recombining or non-recombining using the PHI statistic [46]. Similar to Lassale et al. [7], we found that recombining genes had higher GC content than non-recombining genes, though this difference was small (paired t-test, df = 154, p = 1.503 × 10−11; Fig 4). Interestingly, while a

link between recombination and GC content was apparent, it seemed to explain none of the difference between Ku-encoding and Ku-lacking organisms (Fig 4a). In fact the difference in GC content between recombining and non-recombining genes was actually smaller for Ku-encoding organisms than Ku-lacking ones, the opposite of what we would expect if recombination were driving the link between Ku and GC content (t-test, df = 83.698, p = 0.0308; Fig 4b).”

and (Fig 4 legend):

“Recombination contributes to GC content locally but cannot explain the relationship between GC content and Ku incidence. (a) The mean GC content of genes with evidence for recombination (PHI statistic, [46], see methods) plotted against the mean GC content of genes without evidence for recombination in a given closely related cluster of organisms (ATGC databse [43]). Recombining genes have slightly higher GC content than non-recombining genes (points mostly lie above the dashed x = y line). (b) The difference in GC content for recombining and non-recombining genes within a cluster is smaller for Ku-encoding than Ku-lacking clusters. Clusters classiffed as Ku-lacking if no members encoding Ku (n = 114) and Ku-encoding if at least one member has Ku (n = 41). Clusters excluded if no evidence for recombination was found for any of their genes.”

See methods for details (lines 483-493):

“We obtained all available alignments of shared genes within each cluster of organisms in the ATGC database ([43]). We then ran the program PhiPack [46] using 10000 permutations to generate p-values for the occurrence of recombination in each cluster-gene pair. To correct for multiple testing we used a Benjamini-Hochberg correction with a false-discovery rate of 5%. Altogether this yielded 52117 genes with significant evidence of recombination out of 438580 cluster-gene pairs with sufficient information to run PhiPack. To obtain GC content for each cluster-gene pair we took the mean GC content across sequences in the relevant alignment. To obtain cluster-wide estimates of GC content and Ku incidence we took the mean across genomes associated with organisms in that cluster (each cluster member in ATGC is associated with a RefSeq genome).”

L216-220: “the extremely strong and specific association between GC and Ku suggests that this relationship may be particular to the specific conditions selecting for Ku (especially considering the absence of an association between HR and GC when looking between genomes [5]; S7 Fig)”
As discussed above, these datasets are very unlikely to be matched with the authors’, and rejecting the association of elevated %GC (or Ku occurrence) with HR rates on this basis is possibly flawed. Again, I would suggest the authors run their own recombination tests/quantifications on their own datasets so they can draw robust conclusions.

See comments directly above and S10 Fig.

Also, we would like to point out that even Lassalle et al. didn’t find a link between recombination and GC content when looking between genomes, but only when looking within genomes.

L217 “association between GC and Ku”; L219 “association between HR and GC”; L223 “association 223 between NHEJ and high GC content” and more:
The authors need to use a consistent term to refer to the A/T vs. G/C base composition of genomes; the early sections of the manuscript use the acronym ‘%GC’, but later just name it ‘GC’, or ‘GC content’. One term should be chosen and used throughout the manuscript

All instances have been changed to “GC content”.

L223: “Given our lack of enthusiasm for BGC as a mechanism”
I appreciate the author’s willingness to disclose any subjective bias they may have towards one or another scientific hypothesis, but I don’t think it is appropriate to use it to justify what they investigate. Please rephrase into something like “Given the lack of evidence in support of the BGC hypothesis as reported above, we chose to investigate an alternative hypothesis.”
Importantly, the authors should make clear that they are not opposing hypotheses, i.e. rejecting BGC because of support for the selection hypothesis, or vice versa. In principle, both hypotheses could be true, and so could be a third (or more) alternative that was not yet proposed in the literature.

Changed as requested, now using Fig 4 as a motivation (lines 262-263):

“Given the inability of BGC to explain the association between NHEJ and high GC content (Fig 4), perhaps selection can provide an alternative hypothesis.”

L228-229: “high GC content may promote DNA repair, both by facilitating canonical NHEJ 228 (i.e., Ku-dependent) and alternative NHEJ (i.e., Ku-independent) pathways.”
Please cite relevant literature supporting these claims. If they are supported by the references [50, 51, 52] cited in the following paragraph, please connect these text sections (e.g. by not ending the sentence L229 and connecting it to the next with a colon) so to make it clear.

References added and clarified as requested (lines 266-268):

“In fact, high GC content may promote DNA repair, both by facilitating canonical NHEJ (i.e., Ku-dependent [51, 52, 53]) and alternative NHEJ (i.e., Ku-independent [32, 54]) pathways.”

L232-234 “Any factor that stabilizes this interaction (e.g., high GC via an increased number of hydrogen bonds) may thus increase the efficiency of NHEJ repair.”
L238-239: “It stands to reason that high GC content in these regions of microhomology might help stabilize the end-pairings and improve the efficiency of repair.”
Again, please cite the relevant literature (redundancy of citation with the previous sentence is not an issue in my opinion) so to clarify whether this is a (reasonable) speculation of mechanism by the authors or something that is backed by experimental evidence.

We do feel that the requested redundant citation is a bit awkward, but have followed the reviewers suggestion (lines 271-273):

“ Any factor that stabilizes this interaction (e.g., high GC via an increased number of hydrogen bonds) may have the potential to increase the efficiency of NHEJ repair [51, 52, 53].”

and (lines 278-280):

"It stands to reason that high GC content in these regions of microhomology might help stabilize the end-pairings and improve the efficiency of repair [32, 54]."

L235-237: "alternative high-fidelity end-joining pathways that are independent of the NHEJ machinery, and that these pathways are primarily dependent on nearby microhomology to tether the DNA ends together"
Please clarify which bits of sequence are required to present microhomology for the NHEJ or NHEJ-independent end-joining pathways to function. If it is the immediate sequence on both free ends of the broken dsDNA, this means that sequences with short repeats would be more likely to be repaired by these pathways. This would come as a confounding factor for the prediction of effect of %GC in this system (for instance, because short repeats are enriched in mobile elements like phages, transposons or integrons, which are themselves generally AT-rich…); the authors should mention these potential pitfalls as they develop this hypothesis.

Apologies for any confusion here. These homologous stretches are typically short and internal to the DNA ends, meaning this pathway should not be dependent on the existence of short repeats. We have clarified this in the main text (lines 274-278):

"It has also been shown that prokaryotes can employ alternative high-fidelity end-joining pathways that are independent of the NHEJ machinery [32, 54], and that these pathways are primarily dependent on short (2-5bp [32]) nearby microhomology (DNA ends are typically degraded to reveal internal homologies [32, 54, 55]) to tether the DNA ends together.."

L262-264: "We further predicted that, for restriction enzymes with low GC recognition sequences, the bases flanking restriction sites on the genome would have elevated GC"
The test presented afterwards could also support selection-free hypotheses where the converse rationale would stand, i.e. that increased repair at those DSB-prone sites would induce higher %GC; typically, it would be in line with the BGC hypothesis as HR-associated pathways are also taking part in the repair or restriction enzyme-induced breaks.

A fair point, which we now note in the main text (lines 310-314):

"In principle, evidence of high GC content near breaks could also be taken as support for BGC (despite other evidence to the contrary [10, 49]) since the rate of HR repair should increase locally, meaning that ultimately experimental approaches will be needed to tease apart these hypotheses."

Though again we emphasize that other groups have strongly questioned the basis for BGC as a driver of GC content (Bobay and Ochman [10], Liu et al. [49]). BGC also cannot explain Fig 5a (though, again, this evidence is rather indirect and experimental approaches are preferable).

L259: "to help ameliorate the effects of autoimmunity"
'mitigate' instead of 'ameliorate'

Changed.

L316:” This identified 21389 genomes containing Ku out of a 316 total 104297 genomes analysed” Please provide a list of the genomes, and of which were deemed positive for the Ku protein-coding gene.
L322: “we downloaded alignments from the Alignable Tight Genomic Cluster (ATGC) database [43]” Please provide the list of genomes assigned to cluster, the number of gene alignments and clarify how many were dropped/retained when filter were applied.
L324: “Trait data were obtained from the ProTraits microbial trait database (2679 species; [39])” Please provide the table of how genomes from RefSeq were matched with entries of ProTraits (or if sharing identifiers, a list of genomes covered by both databases).

Please see the github for this paper (as requested): https://github.com/jlw-ecoevo/gcku

L382: “The rationale behind this test”
No test has been described at this point; I assume the authors refer to the comparison of the expected %GC (based on the mutational pattern estimated from phased polymorphism data) to the realized genomic %GC, which they describe right after; please rephrase.

Changed (lines 457-458):

“In order to estimate mutational biases, we assume that recent polymorphisms will not have had a chance to undergo selection (or BGC).”

L413: “no genomes with multiple AT-rich enzymes”
Please clarify how you define AT-rich enzymes (if based on the composition of the target sequence, what threshold of %GC?).

We apologize if this was unclear, as it was noted directly before this phrase (>75% AT). We have rephrased the sentence to be clearer (lines 496-499):

“We then restricted our analyses to genomes encoding enzymes that had low-GC content restriction sequences (AT-rich restriction sequences defined as those with ≥ 75% AT, n = 214; no genomes had multiple enzymes with AT-rich targets).

L424: “We then repeated the above”
Please specify how many draws of thee permutations were conducted.

Again, apologies if this was unclear. We simulated only a single dataset, though it is quite large. Repeated simulation might reduce the variance in our estimates of excess GC near restriction sites, but our current approach should be unbiased (i.e., more simulations could give us slightly more power potentially, but the already large size of the dataset makes this unlikely). We have clarified this in the main text (lines 510-512):

We then repeated the above flank-analysis with this set of “fake” restriction recognition sequences (a single, large simulated dataset was generated with 15923 genome-enzyme pairs)”
